# Application of Hyperspectral Imaging to Detect *Sclerotinia sclerotiorum* on Oilseed Rape Stems

**DOI:** 10.3390/s18010123

**Published:** 2018-01-04

**Authors:** Wenwen Kong, Chu Zhang, Weihao Huang, Fei Liu, Yong He

**Affiliations:** 1 School of Information Engineering, Zhejiang A & F University, 666 Wusu Street, Hangzhou 311300, China; wwkong16@zafu.edu.cn; 2 College of Biosystems Engineering and Food Science, Zhejiang University, Hangzhou 310058, China; chuzh@zju.edu.cn (C.Z.); whhuang@zju.edu.cn (W.H.); yhe@zju.edu.cn (Y.H.); 3 Key Laboratory of Spectroscopy Sensing, Ministry of Agriculture, Hangzhou 310058, China

**Keywords:** oilseed rape stem, *Sclerotinia sclerotiorum*, second derivative spectra, discriminant models

## Abstract

Hyperspectral imaging covering the spectral range of 384–1034 nm combined with chemometric methods was used to detect *Sclerotinia sclerotiorum* (SS) on oilseed rape stems by two sample sets (60 healthy and 60 infected stems for each set). Second derivative spectra and PCA loadings were used to select the optimal wavelengths. Discriminant models were built and compared to detect SS on oilseed rape stems, including partial least squares-discriminant analysis, radial basis function neural network, support vector machine and extreme learning machine. The discriminant models using full spectra and optimal wavelengths showed good performance with classification accuracies of over 80% for the calibration and prediction set. Comparing all developed models, the optimal classification accuracies of the calibration and prediction set were over 90%. The similarity of selected optimal wavelengths also indicated the feasibility of using hyperspectral imaging to detect SS on oilseed rape stems. The results indicated that hyperspectral imaging could be used as a fast, non-destructive and reliable technique to detect plant diseases on stems.

## 1. Introduction

Crop diseases are major threats to crop growth, resulting in crop yield and quality loss [1,2,3]. Timely and proper disease control is crucial for crop safe and efficient production. *Sclerotinia sclerotiorum* (SS) is one of the most serious diseases on oilseed rape. It can infect all aboveground parts of the oilseed rape plants, including leaves, stems, flowers and pods [4,5]. Stems are the most sensitive parts to SS, which also cause the most severe consequences. The ascospores of SS are generated from the apothecia in the soil or the seeds. Treating soil and seeds with fungicides is the most effective method to prevent SS. However, due to the complicated structure of soil, and the fact that some of ascospores are dispersed more widely from other fields into surrounding crops, full prevention of SS on oilseed rape is quite difficult. 

Early detection, timely prevention and control of SS on oilseed rape plants provide another effective method for disease control. The major issues then come to how to effectively and accurately detect SS on oilseed rape plants. Traditional methods, including DNA, RNA and serological based ones, are the most commonly used methods in disease detection. However, the above methods are laborious, time consuming and requiring complex sample preparation. These methods cannot be used in fields either to conduct rapid on-line, large-scale detection [6,7,8,9].

Rapid and accurate detection of crop diseases in large-scale field is essential for disease control at early stage of infection. Imaging and spectroscopy techniques have been used to detect crop diseases as rapid and accurate methods, and their results were appealing with good detection accuracies. Hyperspectral imaging, known as a technique integrating spectroscopy and imaging, shows the advantage of acquiring the spectral and image information simultaneously. For each pixel, there is a spectrum in the spectral range; for each wavelength, there is a grey-scale image. The advantage of hyperspectral imaging makes it feasible to locate infected areas by applying the detection models on the pixels. 

Stems play an important role in plant growth. Plant diseases on stems can cause serious consequences. Prior to stems, leaves are more frequently used for disease detection [10,11,12]. A fact should be considered that once the stems are infected, the situation will be serious. Sometimes when the infected leaves can be detected, the diseases on stems have become serious. Fast and accurate detection of plant diseases on stems is demanded and of great practical value.

Chemometric methods, including preprocessing, feature selection and modelling methods, are essential in data process of hyperspectral images. Many chemometric methods have been developed based on different principles [13,14,15,16,17]. Robustness and applicability of chemometric methods are essential for real-world application of hyperspectral imaging technique. Selection of optimal chemometric method can lead to better results. Generally, the optimal chemometric methods are selected based on the performances of the methods. Since the comparison is conducted on a single sample set with limited samples [18,19,20,21], the effectiveness of chemometric methods cannot be evaluated. The methods should also be studied in different sample sets to evaluate the robustness and applicability.

Plant stem disease detection as well as robustness and applicability of chemometric methods for data analysis are important problems to be addressed in plant disease detection using hyperspectral imaging. The objective of this study was to explore the feasibility of using hyperspectral imaging to detect and locate SS on oilseed rape stems. The specific objectives were as follows: (1) to explore the efficiency of hyperspectral imaging by comparing the results of two sample sets; (2) to explore the efficiency of the optimal wavelengths of two sample sets; (3) to form visual prediction maps of the infected stems.

## 2. Materials and Methods

### 2.1. Sample Preparation

Oilseed rape (*Brassica napus* L., cv. ZS758) seeds were sown into the seedbed at the experimental farm of Zhejiang University (Hangzhou, China). The seedlings were transplanted into the experimental fields a month later at the 5-leaf stage. The temperature and humidity of growth environment were around 16 °C and 70%. After four months, the oilseed rape stems were suitable for experiments. Two experiments were conducted. For the first experiment, 150 stems were collected without leaves and branches, 60 stems were used as healthy samples and 90 stems were used for SS inoculation. The collected stems were then placed in pallets with distilled water to keep the stems fresh. *Sclerotinia sclerotiorum* were cultured in a potato agar for three days before the stem collection. After the stem collection, the mycelial pellets were selected and inoculated onto the stems. The oilseed rape stems were kept in a controlled environment with a temperature of 20 °C and a humidity of 80%. Forty-eight hours later, 60 healthy stems and 60 infected stems were collected for hyperspectral images acquisition. The second experiment was conducted 48 h after the SS inoculation of the first experiment. The procedure of the second experiment was the same as that of the first one.

### 2.2. Hyperspectral Image Acquisition and Calibration

A hyperspectral imaging system covering the spectral range of 384–1034 nm was used to acquire hyperspectral images of stems. The system was formed by an imaging spectrograph (ImSpector V10E; Spectral Imaging Ltd., Oulu, Finland), coupled with a CCD camera (C8484-05, Hamamatsu, Hamamatsu City, Japan). The major parameters of this hyperspectral imaging system are that the spectral resolution is 2.8 mm, the pixel size is 6.45 μm × 6.45 μm and the frame rate is 8.9FPS. The operating temperature of the camera is 0–40 °C. The environment temperature was around 20 °C when the images were collected. The illumination light was provided by two 150 W tungsten halogen lamps (Fiber-Lite DC950 Illuminator; Dolan Jenner Industries Inc., Boxborough, MA, USA). The hyperspectral imaging system conducted line scanning, and a conveyer belt driven by a stepper motor (Isuzu Optics Corp, Hsinchu, Taiwan) was used to move the samples to be scanned. 

The hyperspectral images acquisition was controlled by the Spectral Image-V10E software (Isuzu Optics Corp). The system was adjusted to be suitable to acquire the clear and non-deformable images, and the height between the lens and the sample, the moving speed of the conveyer and the exposure time of the camera was set as 40 cm, 2.05 mm/s and 0.13 s respectively.

The acquired hyperspectral images should be corrected from raw images to reflectance images by the white reference image and dark reference image. The correction was conducted according to the following equation:

(1) $$IR=\frac{I_{raw}-I_{d}}{I_{w}-I_{d}}$$

where *I_R_* was the corrected image, *I_raw_* was the raw acquired image, *I_w_* was the white reference acquired by the special white Teflon tile with nearly 100% reflectance, *I_d_* was the dark reference image acquired by turning off the light source together with covering the camera lens completely for nearly 0 reflectance.

### 2.3. Spectra Extraction

Two different procedures for spectral data extraction were applied. The first one was to extract the average spectrum of each entire stem for analysis. This procedure was the most widely used spectra extraction procedure in hyperspectral images. The second one was to extract pixel-wise spectra for analysis. The pixel-wise spectra of healthy stems and the infected regions within the infected stems were extracted. 

As in many other studies, the average spectrum of each sample was acquired without preprocessing of pixel-wise spectra [22,23,24]. Average spectra were extracted by averaging pixel-wise spectra of all pixels, and the averaged spectra in many studies showed no absolute random noises due to the average of thousands to hundreds of thousands pixel-wise spectra [22,23]. However, the pixel-wise spectra showed obvious noises. To use pixel-wise spectra for analysis, pixel-wise spectra should be preprocessed to reduce noises. In this study, 2000 healthy pixels and 2000 pixels of infected regions were extracted of each sample set, and randomly divided into the calibration and prediction set at the ratio of 3:1.

### 2.4. Multivariate Analysis

#### 2.4.1. Discriminant Models

To accurately detect SS on oilseed rape stems, pattern recognition methods, including PLS-DA, SVM, RBFNN and ELM, were applied to build discriminant models. 

PLS-DA is a supervised pattern recognition method. PLS-DA is conducted in the same manner as PLS regression (PLSR). PLSR or PLS-DA has great ability to explore the linear relationship between the independent variable and the dependent variables [25,26], especially when the number of the independent variables is greater than the number of dependent variables. PLSR uses numerical variable as dependent variable Y, whereas PLS-DA uses categorical variables. The input of dependent variable of PLS-DA is category values, and the output is numerical due to the regression procedure, thus a threshold value should be set to determine which category the sample belongs to [27,28].

SVM is a widely used supervised pattern recognition method. SVM transforms the original data into a high dimension space, and constructs a hyperplane or sets of hyperplane to maximize the distance of samples from different categories. Kernel functions are essential in SVM to map the original data into a high dimension space. Many kernel functions have been proposed, and radial basis function (RBF) is a widely used and efficient kernel function. In this study, RBF was used as the kernel function of SVM. To conduct SVM, two parameters should be determined for better classification, including the bandwidth of the RBF (γ) kernel and the penalty coefficient (C) [29]. A grid search procedure was used to select the optimal combination of γ and C.

RBFNN is a feedforward neural network. RBFNN has an input layer, a hidden layer and an output layer. In RBFNN, RBF is used as the activation function, and nonlinear transformation of the data from the input space to the output space using the linear combination of RBF is utilized in the network. RBFNN has the advantages of fast learning speed, high generalization ability and arbitrary approximation [30,31]. To conduct RBFNN on Matlab, a spread value should be determined and optimized.

ELM is an emerging learning neural algorithm. ELM contains one hidden layer and one linear output layer, and the weights between the hidden layer and output layer are selected by minimal norm least square method. Different from traditional learning algorithms, ELM can be trained much faster. Because of its excellent performance in classification and regression problems, ELM has been used in many researches of hyperspectral images [32].

#### 2.4.2. Optimal Wavelength Selection

Optimal wavelength selection is quite efficient in spectral data analysis to reduce the collinearity and redundancy of spectra data. The selection of wavelengths carrying the most information could reduce the influence of the uninformative wavelengths, reduce the data amount and improve the model performances. In this study, second derivative spectra and the PCA loadings were used to select optimal wavelengths. 

Second derivative is a widely used spectra preprocessing method. Derivative of spectra could help to improve the spectral resolution and identify the spectral peaks. The spectral peaks of the raw spectra could be highlighted in second derivative spectra (2nd derivative spectra), even quite small peaks. The spectral peaks refer to the typical chemical bonds information, thus these peaks could be selected and used to predict the quality parameters of the samples. Second derivative spectra of different sample categories could be used to identify the differences of the typical chemical bonds. The spectral peaks of 2nd derivative spectra with larger differences could be selected as the optimal wavelengths to discriminant sample categories [33,34].

PCA is a widely used qualitative analysis method in spectral data. PCA linearly transforms the original data variables into new orthogonal variables (called principal components, PCs). The new variables are ranked by the data variances, and the first few PCs contain the most of the useful information and explain most of the total variance. Loading vector of each PC represents the regression coefficients of each wavelength at the corresponding PC, indicating importance of the corresponding wavelengths. The peaks and valleys of the first few PCA loading plots could be manually selected as the optimal wavelengths [35,36]. 

### 2.5. Localization and Visualization of Infected Region

Accurate detection of the stems infected by SS could help to treat the disease, which is quite important in oilseed rape disease control, and reduce the use of fungicides. Furthermore, exact and precise treatment of SS on stems needs to know the location of the infected area. Due to the advantage of acquiring spectral and spatial information simultaneously by hyperspectral imaging, the pixels within the stem could be classified to healthy and infected by the classification models. A distribution map formed by the prediction of the pixels could provide direct visual presentation of the infection, and could help to achieve point to point treatment. The general procedure of image visualization is to apply the calibration models using the optimal wavelengths to predict the pixels within the hyperspectral image. The optimal wavelengths and the calibration models are essential for obtaining good distribution maps. 

### 2.6. Software and Model Evaluation

The cut of hyperspectral image to isolate each stem as individual hyperspectral images was manually conducted on ENVI 4.6 (ITT, Visual Information Solutions, Boulder, CO, USA). The spectral data extraction was conducted on Matlab R2010b (The Math Works, Natick, MA, USA). The image visualization was also conducted on Matlab R2010b. PCA, 2nd derivative preprocessing and PLS-DA was performed on Unscrambler^®^ 10.1 (CAMO AS, Oslo, Norway). The performances of the discriminant models were evaluated by the classification accuracy of the calibration set and the prediction set.

## 3. Results

### 3.1. Sample Average Spectra Analysis

#### 3.1.1. Spectra Features

**Figure 1:**
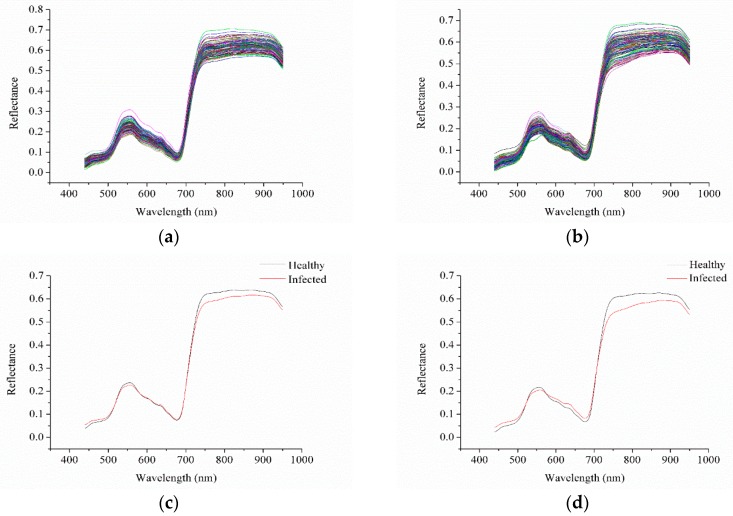
Spectra of sample set 1 and 2 (**a**,**b**) and corresponding average spectra of healthy and infected stems of sample set 1 and 2 (**c**,**d**).

The spectra of healthy and infected stems were acquired in the range of 384–1034 nm. Considering the obvious noises in the head and end of the spectra, only the spectra of 439.89–950.13 nm were analyzed. Moving average smoothing (MAS) with seven smoothing points was applied to preprocess the spectra of the two sample sets. The spectra of sample set 1 and sample set 2 were similar, and it could be observed that the spectral profile of oilseed stems were quite similar to that of oilseed rape leaves [37,38]. The similarity of the spectra between the stems and the leaves were mainly attributed to the pigments. No significant differences were observed in Figure 1a,b. The average spectra of the healthy stems and the infected stems of the two sample sets are shown in Figure 1c,d. Slight differences in the reflectance were observed, especially in the range of 750–900 nm.

#### 3.1.2. Discriminant Models Using Full Spectra

The partial least squares-discriminant analysis (PLS-DA), radial basis function neural network (RBFNN), extreme learning machine (ELM) and support vector machine (SVM) models were built using the full spectra to evaluate the discriminant performances. The results of the discriminant models are shown in Table 1. The PLS-DA, RBFNN, ELM and SVM models of the two sample sets obtained good performances, with the classification accuracies of the calibration and prediction set equal to or higher than 87.5%. The ELM models performed best among all discriminant models. Differences could be observed from the performances of the same model between two different sample sets. Differences could also be observed from the performances of different models for the same sample set. High classification accuracies by different discriminant models of different sample sets indicated that hyperspectral imaging could be used to detect SS on oilseed rape stems. Use of two different sample sets validated the feasibility and potential.

#### 3.1.3. Optimal Wavelength Selection

**Figure 2:**
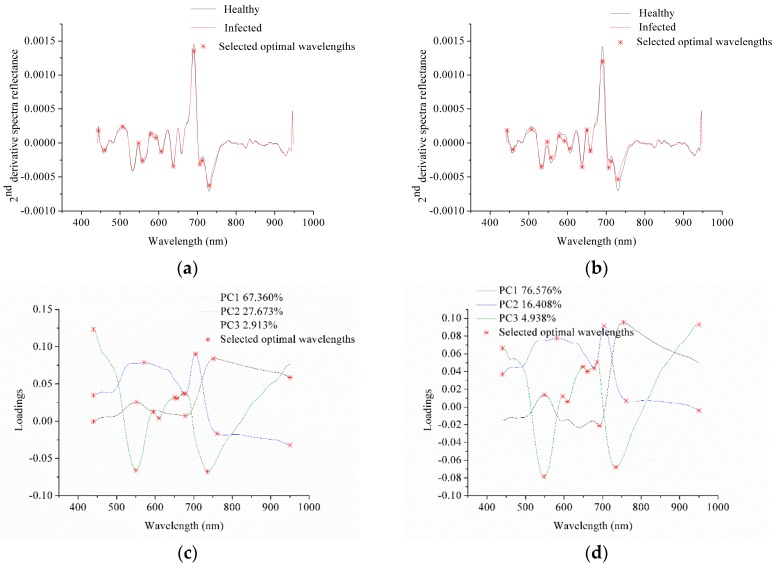
Optimal wavelength selection of sample set 1 and 2 by 2nd derivative spectra (**a**,**b**) and the PCA loadings (**c**,**d**).

Optimal wavelength selection was applied to select a few wavelengths carrying the most useful information to best discriminate healthy and infected stems. Herein, second derivative spectra (2nd derivative spectra) and principal component analysis (PCA) loadings were used to select optimal wavelengths. Average spectra of healthy and infected leaves were used to perform second order Savitzky-Golay (SG) derivative. The first 3 PCs of the two sample sets both explained over 97% of the total variances, and loadings of the first three PCs were used to select the optimal wavelengths. Figure 2 shows the optimal wavelengths selected by 2nd derivative spectra and PCA loadings, and the selected optimal wavelengths are also presented in Table 2.

Figure 2a,b show the optimal wavelengths selected by 2nd derivative spectra. Obvious similarity could be observed from the 2nd derivative spectra of healthy and infected stems between the two sample sets. The peaks and valleys of 2nd derivative spectra of the two sample sets were nearly the same. Optimal wavelengths selected by 2nd derivative spectra were based on the spectral features of healthy and infected samples. Most of the selected optimal wavelengths in the two sample sets were the same or similar. The optimal wavelengths were selected based on the differences in the peaks and valleys of 2nd derivative spectra of healthy and infected stems. The differences in the peaks and valleys of 2nd derivative spectra of healthy and infected stems for the two sample sets were different, resulting in the differences in the selected optimal wavelengths. 

Figure 2c,d shows the optimal wavelengths selected by PCA loadings of the two sample sets. For sample set 1, PC1, PC2 and PC3 explained 67.360%, 27.673% and 2.913% of total variance, respectively. For sample set 2, PC1, PC2 and PC3 explained 76.576%, 16.408% and 4.928% of total variance, respectively. Although each PC between the two sample sets explained different percentage of total variance, the loading lines showed similar shapes.

The selected optimal wavelengths of the two sample sets in Table 2 were similar with slight differences. PCA extracted the useful information of the two sample sets, and the similarity of the two sample sets resulted in the similarity of the optimal wavelengths. Therefore, a general trend could be found that optimal wavelength selection showed good repeatability between different sample sets under the same sample conditions.

#### 3.1.4. Discriminant Models Using Optimal Wavelengths

The PLS-DA, RBFNN, ELM and SVM models using optimal wavelengths of the two sample sets were built. The results are shown in Table 3. All discriminant models obtained satisfactory results, with classification accuracies of the calibration and prediction sets equal to or over 80%. The ELM models performed the best among all models. For each sample set, the same models using optimal wavelengths selected by the two methods showed close results. Different models using optimal wavelengths selected by one method showed significantly different results, indicating the importance of selecting discriminant models. For each optimal wavelength selection method, the same models of different sample sets showed close results, indicating the effectiveness of optimal wavelength selection.

### 3.2. Pixel-Wise Spectra Analysis

#### 3.2.1. Spectral Profile

The use of average spectra of healthy and infected stems provided the potential of fast and accurate detection of SS infected stems. A further study was conducted to locate the infected region for precise detection and control. Knowing the precise location and region of the infected region, the disease control would be more efficient, and the use of fungicides would be minimized. 

**Figure 3:**
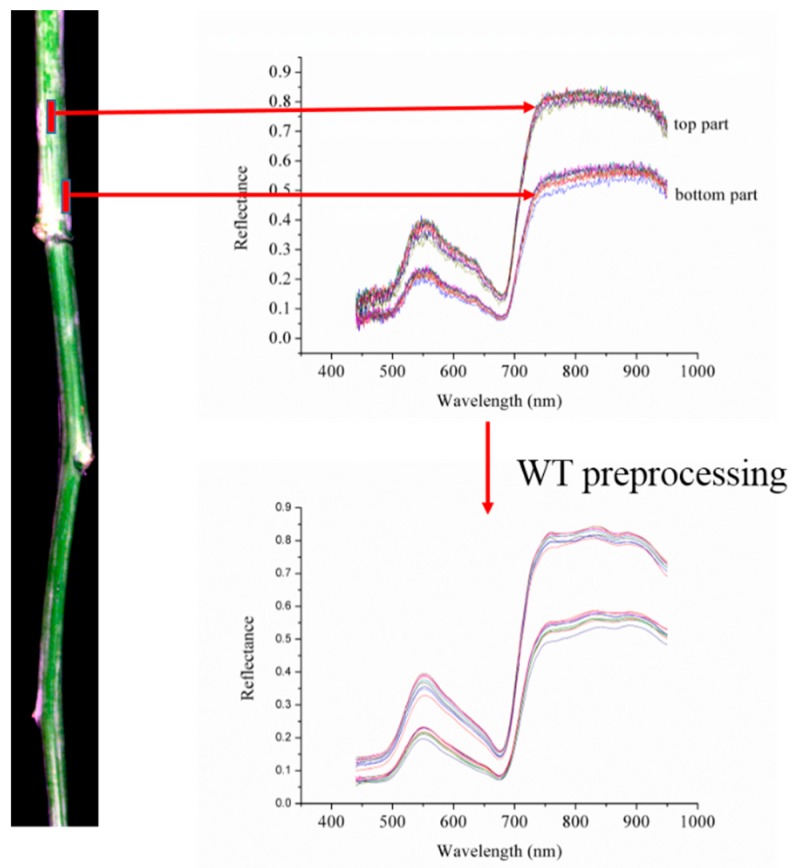
Raw and WT preprocessed spectra of 10 pixels from the middle top and 10 pixels from side bottom of a randomly selected stem of sample set 1.

The pixel-wise spectra were extracted from the healthy stems and the infected region in the infected stems. Considering the obvious noises in the head and end of pixel-wise spectra, only the spectra of 439.89–950.13 nm were analyzed. Pixel-wise spectra (Figure 3) showed obvious noises. 

Wavelet transform (WT) using wavelet function Daubechies 8 and decomposition level 6 was applied to preprocess pixel-wise spectra of sample set 1, WT using wavelet function Daubechies 6 and decomposition level 6 was applied to preprocess pixel-wise spectra of sample set 2 [39]. A fact should be considered that the stem was not flat. Ten pixel-wise spectra of healthy stem from the middle part along with the stem direction and 10 pixel-wise spectra from edge on both sides of the healthy stem sample set 1 are shown in Figure 3. The pixel-wise spectra from the middle part showed obviously higher reflectance than those from the edge part, demonstrating the influence of sample shape. This influence was considered for pixel-wise spectra extraction. Two thousand pixel-wise spectra each of healthy and infected regions were selected for each sample set. Larger differences of spectra could be observed when compared with Figure 1. The reason was that the pixel-wise spectra were extracted from the infected regions, while average spectra were acquired from the uninfected regions and the infected regions. The pixel-wise spectra were divided into the calibration set and the prediction set at the ratio of 3:1.

#### 3.2.2. Discriminant Models Using Full Pixel-Wise Spectra

The PLS-DA, SVM, RBFNN and ELM models were built using the full pixel-wise spectra. The results are shown in Table 1. All discriminant models of the two sample sets obtained good results, with the classification accuracies of the calibration and prediction set over 99%. Classification accuracies of the RBFNN, SVM and ELM models were over 98%, and the PLS-DA model performed relatively worse with the classification accuracies lower than 98%. The same models for the two sample sets obtained similar results, with slightly differences caused by the sample sets. 

#### 3.2.3. Optimal Wavelength Selection for Pixel-Wise Spectra

PCA loadings and 2nd derivative spectra were used to select optimal wavelengths for pixel-wise spectra. Average spectra of pixels from the healthy stems and the infected regions were used to obtained 2nd derivative spectra. For sample set 1, PC1, PC2 and PC3 explained 85.199%, 12.022% and 1.506% of total variance. For sample set 2, PC1, PC2 and PC3 explained 81.493%, 13.694% and 3.279% of total variance. The first 3 PCs of the two sample sets explained more than 98% of total variance, and loadings of the first 3 PCs were used to select optimal wavelengths. 

**Figure 4:**
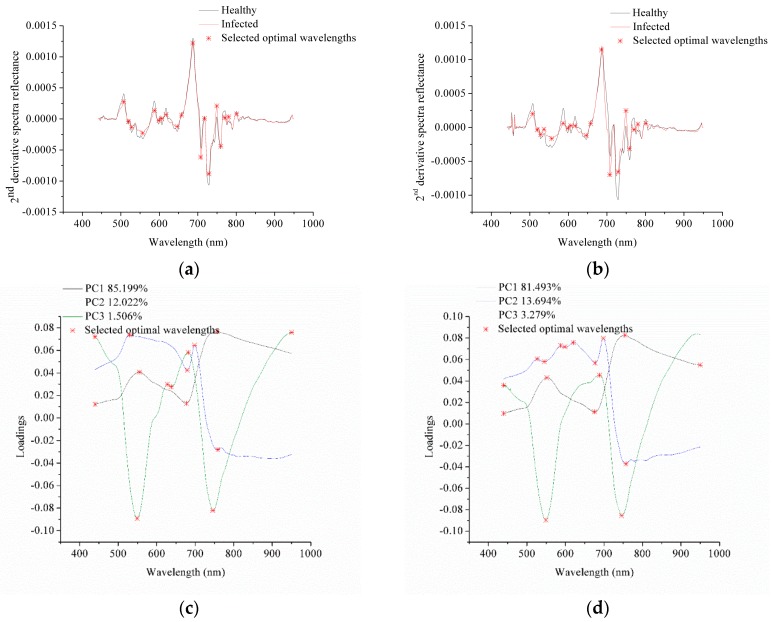
Optimal wavelengths selection of sample set 1 and 2 by 2nd derivative spectra (**a**,**b**) and the PCA loadings (**c**,**d**).

The optimal wavelength selection by 2nd derivative spectra and PCA loadings are shown in Figure 4, and the corresponding selected optimal wavelengths are presented in Table 4. Second derivative spectra of sample sets 1 and 2 showed similar shape, and the selected optimal wavelengths were the same. Although each PC explained different percentage of total variance between the two sample sets, the loading line of each PC showed similar shape with slightly differences caused by the samples. The selected optimal wavelengths by PCA loadings of the two samples were quite close. The results indicated the repeatability of optimal wavelength selection by 2nd derivative spectra and PCA loadings between different sample sets. 

#### 3.2.4. Discriminant Models Using Optimal Wavelengths

The PLS-DA, SVM, RBFNN and ELM models were built using the optimal wavelengths selected by the two methods. All discriminant models showed good discriminant performances with classification accuracies of the calibration and prediction set of the two sample sets over 90% (shown in Table 5). The SVM, RBFNN and ELM models obtained better results, with classification accuracies over 98%, while the PLS-DA model obtained slightly worse results with classification accuracies lower than 97%. For each sample set, the same discriminant models using optimal wavelengths selected by the two different methods showed quite close results. For different sample sets, the same models using optimal wavelengths selected by the same method also obtained quite close results.

#### 3.2.5. Visualization of Infected Regions within the Stem

**Figure 5:**
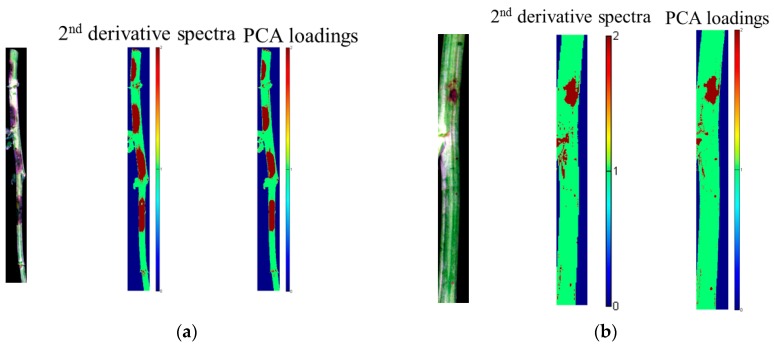
Image visualization of one randomly selected sample from each sample set by the SVM models using optimal wavelengths (**a**) sample set 1; (**b**) sample set 2.

The SVM models using optimal wavelengths selected from pixel-wise spectra of the two sample sets were used to locate the infected regions within the stems. Two randomly selected infected stems of the two sample sets were used for visualization. The same spectral preprocessing was conducted on the spectra of each pixel within the two hyperspectral images. The visualization maps formed by the SVM models using optimal wavelengths selected by 2nd derivative spectra and PCA loadings are shown in Figure 5. The visualization maps matched well with the actual distribution in the two sample sets. The results showed that hyperspectral imaging combined with chemometric methods could locate the infected region effectively. 

## 4. Discussion

A hyperspectral imaging system covering spectral range of 384–1034 nm was used to detect SS on oilseed rape stems. Two sample sets formed by healthy and infected stems were used to explore and validate the feasibility of the system. The overall results indicated that hyperspectral imaging could be used to detect and locate SS on oilseed rape stems.

The feasibility and efficiency of plant disease detection based on leaves have been explored by hyperspectral imaging. However, some important issues needed to be addressed for plant disease detection by hyperspectral imaging. Based on the characteristics of different plant diseases, the influences of plant diseases on different plant tissues varied. Leaves were important tissues in plant growth, leaves were more easily to be infected and sampled than other tissues, and studies focused more on leaves than other tissues. But detection of plant diseases on leaves was not enough, especially when the disease firstly infected other tissues and caused more serious consequences in other tissues. In this study, the oilseed rape stems infected by SS caused the most serious consequences, thus detection of plant diseases were needed. The studies of other infected tissues of plants lacked and should pay attention to other infected tissues.

Hyperspectral imaging provided average spectra of samples and pixel-wise spectra within samples. Utilization of spectral information was important for hyperspectral imaging application. Average spectra of each sample have been widely used in detecting plant diseases [40]. Use of average spectra of hyperspectral imaging was the same as Vis/NIR spectroscopy. The differences was that the spectra of Vis/NIR spectroscopy were collected from a small part of the leaves [41,42] and each sample has one spectra averaged by several times of scans, while average spectra of hyperspectral imaging was acquired from a predefined region of interest (ROI) in the sample. The use of average spectra of infected samples had two situations, average spectra of the entire sample including the infected region and the healthy region within the sample, and average spectra of only the infected region [40,41]. The former was more effective, samples with or without visible symptoms could be predicted in this situation. For the latter situation, samples without visible symptoms were impossible to be predicted, due to the unknown location of the infected regions.

The use of average spectra would help to rapidly and accurately detect plant diseases. However, the location of infected regions could not be known by the average spectra. Models built by average spectra of just dozens to two hundred of samples may not cover the spectral features of different parts within a sample and the spectral features of unknown samples. Considering the difficulty of acquiring the large number of samples, the possibilities to acquire representative spectra were limited. Pixel-wise spectra within each sample showed features of each sample, including physicochemical properties. Pixel-wise spectra could provide detail information of each part within a sample, while average spectra showed the general information, and some detailed information were missing. The use of pixel-wise spectra could highly extend the range of spectra features. In this study, diameters of different stems were different, and the distances between different parts of a stem and the detector were different, resulting in the great differences on reflectance value. It should be noted that the typical symptoms of SS on oilseed stem were the same among different stems of oilseed rape. Thus, the problem for stem disease detection was to acquire representative spectra to form a spectra database and to overcome the spectra differences caused by the different diameters. 

The prediction maps formed by pixel-spectra based models showed that pixel-wise spectra were effective for plant disease detection. A problem in bringing hyperspectral imaging to real-world application was that it was quite difficult to obtain representative spectra from samples. Pixel-wise spectra provided an alternative to obtain representative spectra from samples. Take oilseed rape stems in this study for example, stems infected by SS showed similar symptoms, and the major difference in shape was the diameters. Different stems might have different diameters, and the distance between different parts of a stem and the detector was different. Pixel-wise spectra of healthy and infected pixels in different parts covered spectral features relating to sample shapes and symptoms. Thus, a pixel-wise spectrum could be used to present the spectral features of the pixels in different stems which had the same distance between the pixel and the detector. Hence, there was no need to seek a lot of samples to search for the representative spectra. A representative spectra database was feasible by using pixel-wise spectra. As in this study, pixel-wise spectra extended the spectral features and could be used to locate the infected regions in stems.

After selecting representative spectra, chemometrics was another essential important issue to be addressed. To bring hyperspectral imaging to real-world application, qualitative analysis of spectral features was not enough, discriminant models should be built [42,43]. Without robust and accurate models, real-world application of hyperspectral imaging was impossible to achieve. Detection of plant diseases was still at the research stage [44], the use of chemometric methods has not been fully explored. There were many discriminant methods, some models obtained acceptable results, and they performed differently. In general studies, discriminant models were used in one sample set, whether the discriminant models could also be used in other sample sets or unknown samples needed to be studied. In this study, the same discriminant models obtained different results for two different sample sets, and the models with the best performances were different in the two sample sets. Therefore, it was difficult to conclude which model was the best for real-world application. However, a general trend could be found from different sample sets. Optimal discriminant models with greater applicability and universality should be developed. Moreover, discriminant models using pixel-wise spectra all showed satisfactory results in different sample sets, showing the effectiveness of pixel-wise spectra. Models using more representative spectra could be more effective.

One other problem for hyperspectral imaging was that the large amount of data and the high cost of hyperspectral imaging. Optimal wavelength selection was quite important in hyperspectral imaging, which could result in significant reduction of data amount and improvement of modeling efficiency. Multi-spectral imaging system could be developed using the selected optimal wavelengths, which could significantly reduce the instrument costs. Selecting optimal wavelengths with great universality and repeatability among different samples was essential for these purposes. According to previous studies, optimal wavelengths selected by different methods were different [45,46]. However, some of the optimal wavelength selection methods were based on performances of discriminant models. For example, wavelengths selected by PLS (PLS regression or PLS-DA) based optimal wavelength selection methods highly depended on the performances of the PLS models [47,48]. Different optimal LVs and performances of the PLS-DA models in the two sample sets could be found, which would affect optimal wavelengths selection. Optimal wavelengths selected by weighted regression coefficient (*Bw*) are presented in Table 6 to show that the selected optimal wavelengths were different in different sample sets. Selection of optimal wavelengths based on spectral features of the samples would help to avoid the influence of model performances. Second derivative spectra for optimal wavelength selection were based on the presented spectral peaks, and the PCA loadings for optimal wavelength selection were based on the useful information of the spectra. It could be found that although discriminant models showed different performances, optimal wavelengths selected by the two methods were similar in the two sample sets for average spectra and pixel-wise spectra. The use of 2nd derivative spectra and PCA loadings showed great potential in selection optimal wavelengths with universality and repeatability in different sample sets, which showed great potential to develop low-cost on-line multi-spectral imaging system for practical applications.

Besides, the results of visualization showed the great potential of hyperspectral imaging for early detection and localization of disease infection when there were no visible symptoms, which could not be rapidly, noninvasively and accurately detected by other techniques. The prediction maps would provide visual information for plant disease location and regions. Knowing the infected regions in hyperspectral images would help to evaluate the disease severity by identifying pixels of infected regions and the sample regions. The use of hyperspectral imaging would provide great benefits in crop disease detection and control.

In all, hyperspectral imaging as a rapid and nondestructive technique showed great potential in plant diseases detection. Along with the technology development, the acquisition equipment would be easier to carry and operate. It will provide not only canopy level but more information in different levels. To bring hyperspectral imaging to real-world application, it was important to extend the research from leaves to other tissues, to develop discriminant models with great universality for real-world application and to select optimal wavelengths with great universality to reduce data amount and develop low-cost multi-spectral imaging system. The results in this study could provide valuable guidance for bringing hyperspectral imaging to real-world application of plant diseases detection and control.

## 5. Conclusions

Hyperspectral imaging combined with chemometrics was applied to detect SS on oilseed rape stems. Average spectra of healthy and infected stems as well as pixel-wise spectra of healthy stems and infected regions within infected stems of two sample sets were extracted and studied. Optimal wavelengths selected by PCA loadings and 2nd derivative spectra were similar between two sample sets, indicating the effectiveness of optimal wavelengths selection by PCA loadings and 2nd derivative spectra. PLS-DA, SVM and RBFNN models using full spectra and optimal wavelengths of average spectra and pixel-wise spectra for two sample sets all obtained satisfactory detection results, indicating that hyperspectral imaging was a promising technique to detect SS on oilseed rape stems. The use of discriminant models and optimal wavelengths selection methods in two different sample sets indicated that chemometric methods were important for hyperspectral imaging application, and selection of optimal discriminant models and optimal wavelengths selection methods which would obtain good results in different sets was important and would help to bring hyperspectral imaging to real-world application. In future studies, more samples and more chemometric methods will be studied under different situations for using hyperspectral imaging to detect SS on oilseed rape stems, as well as other organs and other crops.

## Figures and Tables

**Table 1:** Results of discriminant models using full spectra and pixel-wise spectra of sample set 1 and 2.

	Models	Sample Set 1			Sample Set 2		
		par ^a^	cal ^b^	pre ^c^	par	cal	pre
Average spectra	PLS-DA	9	98.75	100	10	98.75	92.50
	RBFNN	0.8	100	97.50	0.1	100	87.50
	ELM	20	100	100	19	100	97.50
	SVM	(16, 1.7411)	97.50	92.50	(9.1896, 0.0206)	97.50	90.00
Pixel-wise spectra	PLS-DA	8	95.43	94.80	8	97.53	96.60
	RBFNN	0.4	100	98.80	0.6	100	98.70
	SVM	(256, 0.0039)	99.27	99.00	(84.4485, 0.0118)	99.73	99.30
	ELM	367	99.83	99.40	416	99.77	99.50

^a^: par represents the parameters of the models, meaning number of optimal latent variables (LV) in PLS-DA model, spread value in RBFNN model, number of nodes in the hidden layer in EKM model, (C, γ) in SVM model; ^b^: cal represents the calibration set (%); ^c^: pre represents the prediction set (%).

**Table 2:** Optimal wavelengths selection of sample set 1 and 2 selected by 2nd derivative spectra and the PCA loadings of sample set 1 and 2 using sample average spectra.

Method	Sample Set 1		Sample Set 2	
	Number	Wavelength (nm)	Number	Wavelength (nm)
2nd derivative spectra	13	443.53, 460.58, 507.12, 547.86, 557.78, 578.91, 592.63, 606.38, 637.75, 690.83, 706.69, 712.45, 730.32	16	443.53, 459.36, 507.12, 533.01, 547.86, 557.78, 578.91, 592.63, 606.38, 637.75, 650.34, 659.18, 690.83, 706.69, 712.45, 730.32
PCA loadings	15	439.89, 550.34, 551.57, 571.44, 595.13, 610.13, 650.34, 656.65, 673.08, 678.15, 704.81, 735.43, 752.08, 761.06, 950.13	16	439.89, 547.86, 549.1, 581.4, 596.38, 608.88, 649.08, 660.44, 678.15, 685.76, 692.1, 703.54, 734.15, 754.65, 761.06,950.13

**Table 3:** Results of discriminant models using optimal wavelengths of sample set 1 and 2.

		2nd Derivative Spectra			PCA Loadings		
		par	cal	pre	par	cal	pre
Sample set 1	PLS-DA	6	93.75	80.00	6	96.25	82.50
	SVM	(1.7411, 0.1895)	95.00	82.50	(48.5029,0.3299)	97.50	80.00
	RBFNN	28	100	97.50	11	100	97.50
Sample set 2	PLS-DA	7	95.00	95.00	9	98.70	92.50
	SVM	(3.0314,5.2780)	97.50	82.50	(5.2780,3.0314)	98.75	87.50
	RBFNN	31	100	92.50	16	100	95.00

**Table 4:** Optimal wavelengths selected by 2nd derivative spectra and the PCA loadings of sample set 1 and 2 using pixel-wise spectra.

Methods	Sample Set 1		Sample Set 2	
	Number	Wavelengths (nm)	Number	Wavelengths (nm)
2nd derivative spectra	19	507.12, 519.44, 528.07, 556.54, 587.64, 598.87, 606.38, 617.65, 646.56, 657.92, 687.02, 707.36, 717.55, 729.04, 749.52, 759.78, 770.06, 780.36, 801.01	19	507.12, 519.44, 528.07, 536.72, 556.54, 586.39, 598.87, 606.38, 617.65, 646.56, 657.92, 685.76, 707.36, 729.04, 749.52, 758.5, 770.06, 780.36, 801.01
PCA loadings	14	439.89, 529.31, 549.1, 555.29, 627.69, 637.75, 676.88, 679.42, 681.95, 698.45, 745.67, 757.21, 758.5, 950.13	16	439.89, 526.84, 545.38, 549.1, 551.57, 587.64, 598.87, 620.42, 675.62, 676.88, 688.29, 698.45, 745.67, 754.65, 757.21, 950.13

**Table 5:** Results of discriminant models using optimal wavelengths of pixel-wise spectra of sample set 1 and 2.

		2nd Derivative Spectra			PCA Loadings		
		par	cal	pre	par	cal	pre
Sample set 1	PLS-DA	7	94.17	94.60	7	93.93	94.30
	SVM	(256, 9.1896)	99.40	98.20	(84.4485,5.2780)	99.47	98.50
	RBFNN	3	99.37	98.40	1	99.63	99.10
Sample set 2	PLS-DA	7	96.60	96.40	4	95.07	95.50
	SVM	(256,16)	99.87	99.40	(27.8576,9.1896)	99.83	99.20
	RBFNN	4	99.37	98.80	2	99.77	99.30

**Table 6:** Optimal wavelengths selection of sample set 1 and 2 selected by *Bw* using sample average spectra.

Method	Sample Set 1		Sample Set 2	
	Number	Wavelength (nm)	Number	Wavelength (nm)
*Bw*	27	447.18,455.71, 474.02, 512.05, 533.01, 554.05, 585.14, 615.15, 633.98, 651.61, 685.76, 692.1, 701.1, 743.11, 789.39, 794.55, 803.6, 811.36, 825.62, 841.21, 847.72, 851.63, 859.46, 879.06, 898.72, 918.45, 950.13	30	460.58, 461.8, 470.35, 482.58, 500.98, 552.81, 580.16, 602.63, 615.15, 649.08, 664.23, 673.08, 683.22, 690.83, 697.18, 707.36, 716.28, 736.71, 761.06, 785.52, 794.55, 802.3, 806.18, 815.25, 825.62, 851.63, 862.07, 869.9, 898.72, 915.81
